# Prospective Human Validation of Artificial Intelligence Interventions in Cardiology: A Scoping Review

**DOI:** 10.1016/j.jacadv.2024.101202

**Published:** 2024-08-28

**Authors:** Amirhossein Moosavi, Steven Huang, Maryam Vahabi, Bahar Motamedivafa, Nelly Tian, Rafid Mahmood, Peter Liu, Christopher L.F. Sun

**Affiliations:** a Telfer School of Management, University of Ottawa, Ottawa, Ontario, Canada; b University of Ottawa Heart Institute, University of Ottawa, Ottawa, Ontario, Canada; c Marshall School of Business, University of Southern California, Los Angeles, California, USA

**Keywords:** randomized clinical trial, human expert validation, machine learning, deep learning, scoping review

## Abstract

**Background:**

Despite the potential of artificial intelligence (AI) in enhancing cardiovascular care, its integration into clinical practice is limited by a lack of evidence on its effectiveness with respect to human experts or gold standard practices in real-world settings.

**Objectives:**

The purpose of this study was to identify AI interventions in cardiology that have been prospectively validated against human expert benchmarks or gold standard practices, assessing their effectiveness, and identifying future research areas.

**Methods:**

We systematically reviewed Scopus and MEDLINE to identify peer-reviewed publications that involved prospective human validation of AI-based interventions in cardiology from January 2015 to December 2023.

**Results:**

Of 2,351 initial records, 64 studies were included. Among these studies, 59 (92.2%) were published after 2020. A total of 11 (17.2%) randomized controlled trials were published. AI interventions in 44 articles (68.75%) reported definite clinical or operational improvements over human experts. These interventions were mostly used in imaging (n = 14, 21.9%), ejection fraction (n = 10, 15.6%), arrhythmia (n = 9, 14.1%), and coronary artery disease (n = 12, 18.8%) application areas. Convolutional neural networks were the most common predictive model (n = 44, 69%), and images were the most used data type (n = 38, 54.3%). Only 22 (34.4%) studies made their models or data accessible.

**Conclusions:**

This review identifies the potential of AI in cardiology, with models often performing equally well as human counterparts for specific and clearly scoped tasks suitable for such models. Nonetheless, the limited number of randomized controlled trials emphasizes the need for continued validation, especially in real-world settings that closely examine joint human AI decision-making.

Artificial intelligence (AI) is transforming cardiovascular care by enabling the rapid analysis of large data sets to uncover patterns that are not easily detected by human experts.^1^^,^^2^ Accordingly, tools for AI creation, namely machine learning models, offer promising avenues for advancing cardiovascular disease screening, diagnosis, monitoring, and treatment. The combination of increasingly vast data sets, pressures to improve health care system efficiency, and patient demand for personalized care, has accelerated the development of AI in cardiology.^3^ Such AI models have already been developed for atrial fibrillation detection,^4^ heart failure risk prediction,^5^^,^^6^ and ejection fraction estimation.^7^^,^^8^ However, despite these advancements, the integration of AI models into practice has faced significant challenges, limiting their real-world impact.^9^

A fundamental barrier to the adoption of AI is the evidence scarcity evaluating AI models against human experts and gold standard practices.^10^^,^^11^ Such evidence is critical in high-stakes settings such as cardiology, where AI model uptake in practice is nuanced, requiring human-AI collaboration, and sufficient algorithmic trust and model performance.^12^ Therefore, understanding the outcomes of prospective human and real-world validation studies, namely randomized controlled trials (RCTs), pilot implementations, and human-expert benchmarking studies, is essential to building evidence to inform AI use in practice.

In this scoping review, we explore the current evidence regarding the prospective human expert and real-world validation of AI models in cardiology. Distinct from previous reviews that focus on only RCTs of AI models across multiple health disciplines^10^^,^^11^ or discuss the use of AI models in cardiology without emphasizing prospective validation,^13^^,^^14^ our review focuses on the *prospective human validation* of AI models in *cardiology*. This review focuses on: 1) identifying and analyzing AI models based on their comparative performance against human experts in prospective experiments; and 2) evaluating the outcomes of AI models assessed through RCTs and pilot studies, emphasizing their real-world applicability.

## Methods

### Study design

This scoping review followed the Preferred Reporting for Scoping Reviews (PRISMA-ScR) reporting guidelines.^15^ The final protocol was registered with the Open Science Framework on March 15, 2024 (Center for Open Science). Institutional Review Board approval was not required due to the use of publicly available data. The inclusion criteria involved published articles on AI interventions in cardiology that are prospectively validated through the comparison of AI model outputs with human expert or gold standard decisions, RCTs of AI interventions, and pilot implementation of AI models in real-world settings. AI models and interventions include technologies that use machine learning algorithms, such as deep learning, and generative AI models. In the study, included RCTs must comply with the national registration standards to ensure methodological transparency and rigor. Pilot studies refer to implementations or preliminary investigations of AI models to assess impact, feasibility, cost, and risk. Human comparison studies are defined as research that compare outcomes of an AI model against experts in the domain (see Supplemental Methods for full details).

### Search strategy and selection criteria

Both MEDLINE and Scopus online databases were systematically searched. The search was limited to include all English published articles between January 2015 and December 2023. As multiple articles focused on prospective human expert and real-world model validation were accompanied by a prior paper detailing the retrospective training and testing of the AI model, backward citation searches were performed during the data extraction stage, when necessary.

### Data extraction

We extracted data on study objectives, clinical use cases (eg, diseases or tasks targeted by AI), and experiment outcomes (ie superiority or noninferiority of the AI intervention’s predictive performance or efficiency relative to the study controls). Studies were classified into RCTs, pilot implementations, or comparisons with human expert benchmarks (hereinafter denoted as *human comparison*) and identified as multicenter or single-center studies. AI interventions were categorized into 4 intervention types: screening (eg, identifies risk in asymptomatic individuals for early intervention); diagnosis (eg, analyzes symptomatic patients to classify cardiovascular conditions and guide treatment); monitoring (eg, aims to continuously assess for and predict adverse events); and treatment (eg, supports optimized management and therapeutic strategies for patient care in cardiovascular diseases). We also categorized the AI intervention as “AI” (ie, AI model independently used) or “joint AI-human expert” (ie, human assisted by AI).

For AI models, we collected algorithm type, training data set size, data type (eg, images, electronic health record [EHR]), prediction task, and public availability of the model or data. Performance metrics collected varied by task: area under the receiver-operating curve (AUC), sensitivity, and specificity for classification; mean absolute deviation, mean absolute error, mean squared error, normalized Bland-Altman (BA) metrics (see Supplemental Methods for details) and intraclass correlation coefficient (ICC) for regression tasks.

### Statistical analysis

Data were summarized using counts, percentages, medians, and IQRs (the 25th percentile-75th percentile of the metric). The total counts for categorical variables may exceed the total 64 included articles because some articles may be classified into multiple categories. When summarizing the performance metrics of regression models, we focused on the normalized BA ICC, and mean absolute deviation metrics, compared to mean absolute error, and mean squared error as they can be reported in a variety of units, preventing direct comparison.

## Results

### General characteristics

**Figure 1:**
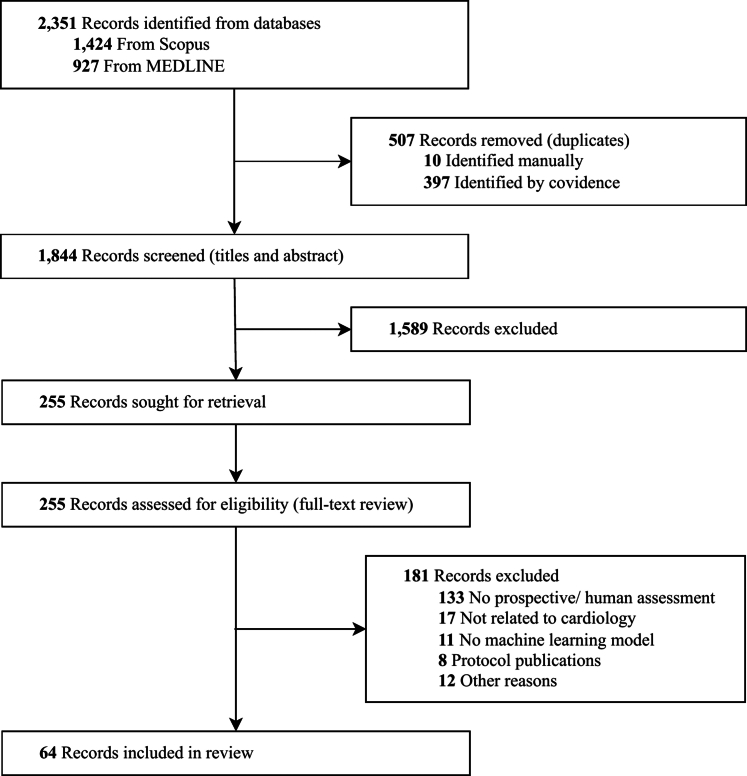
**PRISMA-ScR Flowchart of the Study** The flowchart shows the screening process of the review paper, spanning 2015 to 2023.

A total of 2,351 titles were identified through the search from January 2015 to December 2023 (Figure 1, Central Illustration). The 2 most frequent reasons for full review exclusion were because they did not include prospective/human validation of the AI model and were not related to the field of cardiology. Overall, the studies included for data extraction consisted of 64 published manuscripts.

**Figure 2:**
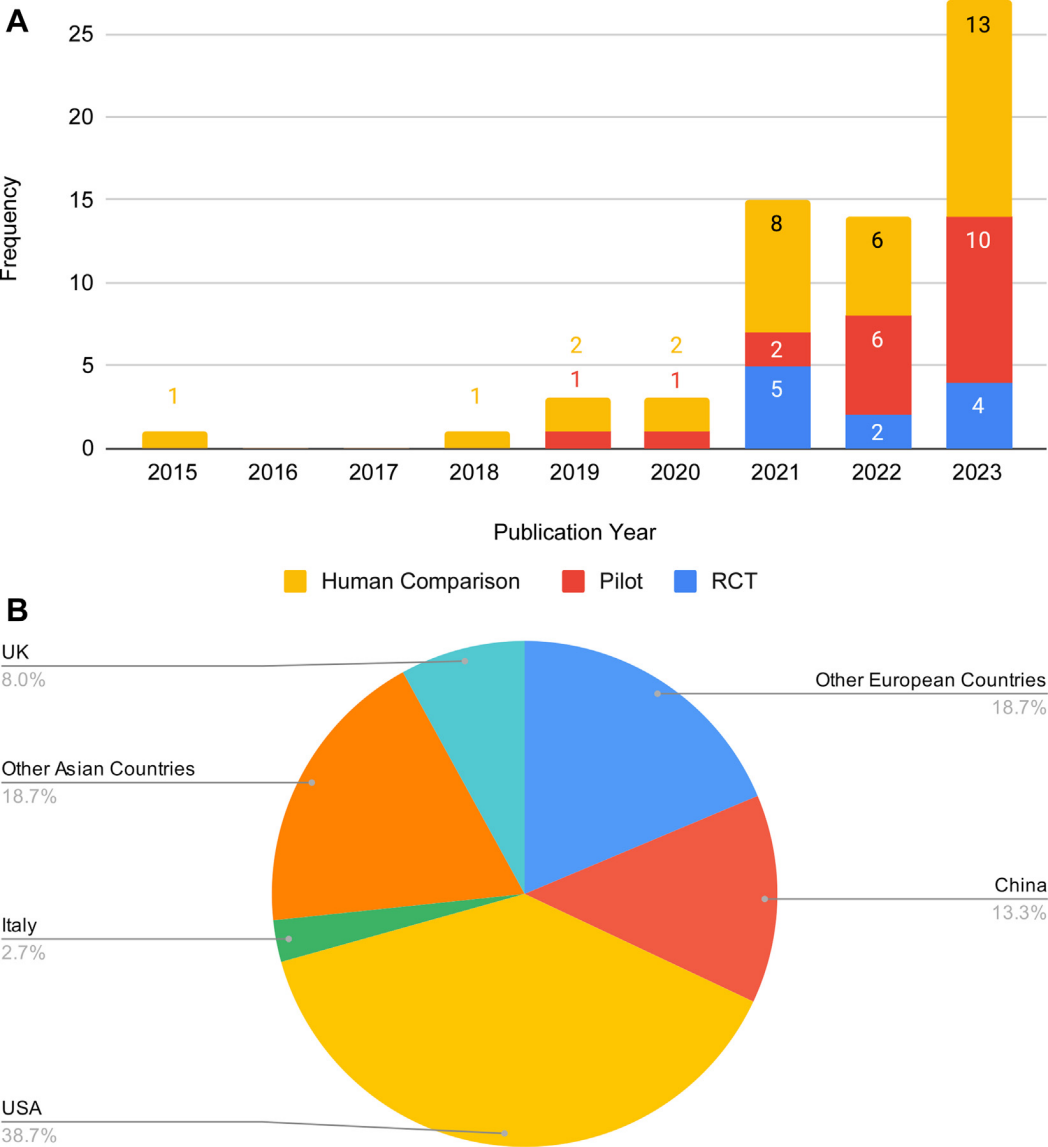
**Temporal and Geographic Distribution** (A) Publication year. (B) Country. A displays the frequency of publications per year from 2015 to 2023, categorized by study type. B shows the proportion of studies by geographic region. RCT = randomized controlled trial.

Approximately 92.2% (n = 59) of the studies were published after 2020, with all 11 RCTs^4^^,^^6^, ^7^, ^8^^,^^16^, ^17^, ^18^, ^19^, ^20^, ^21^, ^22^ published after 2021, indicating an increasing interest and need for prospective validation of AI models (Figure 2A). The majority of studies were from the United States (n = 29, 38.7%) and China (n = 10, 13.3%), underscoring geographic homogeneity in the publications (Figure 2B).

### Study design

**Figure 3:**
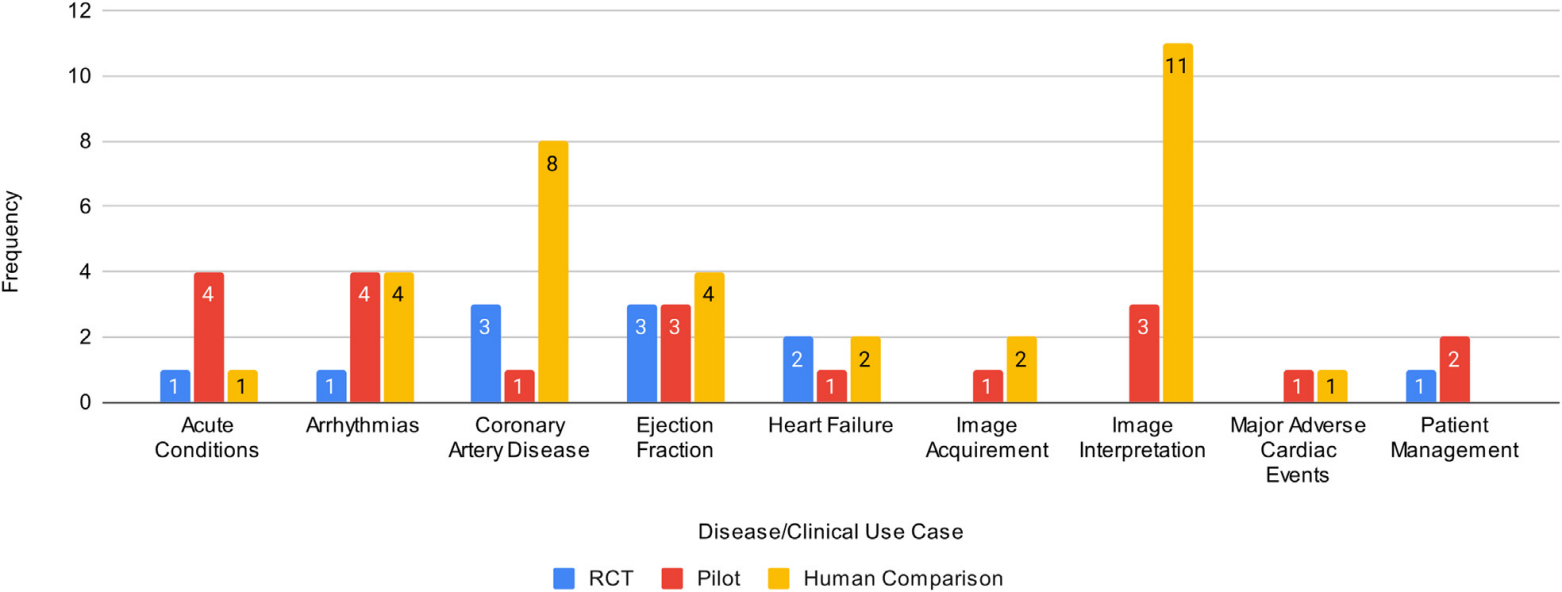
**Frequency of Study Designs by Disease/Clinical Use Case** The number of included studies by design type across the clinical used case categories. Abbreviation as in Figure 2.

Table 1 summarizes the included articles by intervention type, clinical use case, objective, study type, study control, study intervention, and study intervention superiority/noninferiority compared to the control. Most of the articles focus on diagnosis (n = 39, 60.9%), whereas monitoring (n = 6, 9.4%) and treatment (n = 6, 9.4%) have received the least focus. The clinical use cases of the AI tools were diverse, including imaging (eg, computed tomography scans, cardiovascular magnetic resonance, echocardiogram, myocardial perfusion imaging, x-rays) interpretations (n = 14; 21.8%), coronary artery disease (n = 12, 18.8%), ejection fraction measures (n = 10, 15.6%), arrhythmias (n = 9, 14.1%), acute conditions (n = 6, 9.4%), heart failure (n = 5, 7.8%), patient management (n = 3, 4.7%), image acquirement (n = 3, 4.7%), and major adverse cardiac events (n = 2, 3.1%). Figure 3 shows the counts of studies across study design and clinical use cases. We find most RCTs examine ejection fraction (n = 3, 4.7%) and coronary artery disease (n = 3, 4.7%). The pilot studies primarily focused on arrhythmias (n = 4, 6.2%) and acute conditions (n = 4, 6.2%). The human comparison studies were conducted across all categories except for patient management and were most prevalent for image interpretation (n = 11, 17.2%).

**Table 1:** Summary of Study Design and Clinical Characteristics of the Included Studies

First Author Year	Intervention Type	Clinical Use Case	Objective	Study Type	Multicenter	Study Control	Study Intervention	Intervention Superiority/Noninferiority
Blomberg et al 2021.^20^	D	Acute conditions: out-of-hospital cardiac arrest	Examine if ML algorithms can detect out-of-hospital cardiac arrest in 911 calls.	RCT	No	Human	Joint AI-human	Maybe
Huang et al 2021.^4^	M	Arrhythmias: atrial fibrillation	Evaluate a self-applied AI-based ECG device vs traditional follow-up for detecting atrial fibrillation postablation.	RCT	No	Human	AI	Yes
Yao et al 2021.^7^	D	Ejection fraction: ≤35%	Assess AI in early detecting low ejection fraction using 12-lead ECGs.	RCT	Yes	Human	Joint AI-human	Yes
Luštrek et al 2021.^6^	T	Heart failure: congestive	Develop and validate a system for self-managing congestive heart failure.	RCT	Yes	Gold standard	Joint AI-human	Yes
Zisis et al 2021.^16^	T	Heart failure	Examine if an avatar-based heart failure app enhances knowledge, quality of life, and self-care in heart failure patients.	RCT	Yes	Gold standard	AI	Maybe
Rushlow et al 2022.^8^	S	Ejection fraction: ≤35%	Compare and assess the clinicians’ characteristics of "high" vs "low adopters" of an AI-ECG tool alerting for low ejection fraction detection.	RCT	No	Human comparison	Joint AI-human	Yes
Horne et al 2022.^17^	T	Coronary artery disease: statin adherence	Examine if rare, personalized nudges via ML boost statin therapy adherence.	RCT	No	Gold standard	AI	Yes
Sandhu et al 2023.^18^	S	Coronary artery disease: calcium	Examine if coronary artery calcium screening from past chest CT scans and subsequent notifications raise statin prescriptions.	RCT	Yes	Gold standard	Joint AI-human	Maybe
Yang et al 2023.^19^	D	Coronary artery disease: fractional flow reserve	Assess computed tomography-derived fractional flow reverse via ML on stable coronary artery disease with intermediate stenosis.	RCT	Yes	Gold standard	Joint AI-human	Yes
He et al 2023.^21^	D	Ejection fraction	Compare AI initial assessment with sonographer's on cardiologist's final left ventricular ejection fraction interpretation.	RCT	No	Human comparison	AI	Yes
Šribar et al 2023.^22^	M	Patient management: surgical procedure risk	Determine if an ML-guided hypotension prediction index reduces intraoperative hypotensive events in thoracic procedures versus conventional therapy.	RCT	No	Gold standard	Joint AI-human	Yes
Brennan et al 2019.^23^	S	Patient management: surgical procedure risk	Evaluate and compare the usability and accuracy of MySurgeryRisk algorithm vs physicians in predicting postoperative complications.	Pilot	No	Human comparison	Joint AI-human	Yes
Jacobsen et al 2020.^24^	D	Arrhythmias: atrial fibrillation	Evaluate a wearable device in noninvasive atrial fibrillation detection.	Pilot	No	Gold standard	AI	Yes
Liu et al 2021.^25^	D	Acute conditions: myocardial infarction	Validate a DL model for detecting acute myocardial infarction.	Pilot	No	Human comparison	Joint AI-human	Yes
Maille et al 2021.^26^	T	Arrhythmias: QTc interval monitoring	Assess hydroxychloroquine-azithromycin's cardiac safety for COVID-19 via smartwatch ECG and AI to compare QTc prolongation detection with standard ECGs.	Pilot	No	Human comparison	AI	Yes
Winslow et al 2022.^27^	M	Acute conditions: in-hospital cardiac arrest	Evaluate the effect of electronic cardiac arrest risk triage, an ML risk score, on mortality in high-risk adult inpatients.	Pilot	Yes	Gold standard	Joint AI-human	Yes
Bachtiger et al 2022.^28^	S	Ejection fraction: ≤40%	Validate an AI algorithm on a single-lead ECG for point-of-care left ventricular ejection fraction ≤40% screening.	Pilot	Yes	Human comparison	AI	Yes
Yacoub et al 2022.^29^	D	Image interpretation: CT scan	Assess how an AI platform for chest CT analysis affects radiologists' interpretation times in clinical settings.	Pilot	No	Human comparison	Joint AI-human	Yes
Aharon et al. 2022.^30^	T	Patient management: adherence to cardiac rehabilitation programs	Show that personalized AI interventions can boost patient participation in cardiac rehabilitation programs.	Pilot	No	Gold standard	AI	Yes
Zhu et al. 2022.^31^	S	Arrhythmias: atrial fibrillation	Develop and validate a photoplethysomography-based atrial fibrillation detection algorithm, deployed on a smartwatch.	Pilot	No	Human comparison	AI	Yes
Edalati et al. 2022.^32^	D	Image interpretation: CMR	Implement DL models for slice alignment and cardiac shimming in clinical CMR.	Pilot	No	Human comparison	AI	Yes
						Gold standard		
Noseworthy et al. 2022.^33^	M	Arrhythmias: atrial fibrillation	Prospectively use an AI model to monitor for patients with atrial fibrillation.	Pilot	Yes	Human	AI	Yes
						Gold standard		
Mor-Avi et al. 2023.^34^	D	Image acquirement: echo	Assess quality and diagnostic suitability of echocardiographic exams by novices using new AI software.	Pilot	Yes	Human comparison	Joint AI-human	Maybe
Cho et al. 2023.^35^	S	Acute conditions: in-hospital cardiac arrest	Investigate DeepCARS predictive accuracy for in-hospital cardiac arrest and unplanned intensive care unit transfer in general ward patients versus conventional methods.	Pilot	Yes	Gold standard	AI	Yes
Yamaguchi et al. 2023.^36^	D	Ejection fraction	Evaluate if an AI model estimating expert left ventricular ejection fraction read can reduce interinstitutional variability among level 1 readers.	Pilot	Yes	Human comparison	Joint AI-human	Yes
Nurmaini et al. 2023.^37^	D	Image interpretation: echo	Develop and validate a DL model to segment and classify cardiac septal defects in prenatal and postnatal echos.	Pilot	No	Human comparison	AI	Maybe
Dadon et al. 2023.^38^	S	Ejection fraction	Use a handheld ultrasound with AI to measure left ventricular ejection fraction in COVID-19 patients.	Pilot	No	Human comparison	AI	Yes
Celik et al. 2023.^39^	S	Heart failure	Develop a DL model to diagnose heart failure using x-rays.	Pilot	No	Human comparison	AI	Yes
						Gold standard		
Al-Zaiti et al. 2023.^40^	D	Acute conditions: myocardial infarction	Evaluate ML's diagnostic accuracy for ECG diagnosis and risk stratification of occlusion myocardial infarction without ST-elevation myocardial infarction pattern in an observational cohort study.	Pilot	Yes	Human comparison	AI	Yes
						Gold standard		
Mahajan et al. 2023.^41^	S	Major adverse cardiac events	Build and evaluate an ML model to predict postoperative mortality and major cardiac events using preoperative EHR data.	Pilot	No	Gold standard	AI	Yes
Omori et al. 2023.^42^	D	Coronary artery disease: fractional flow reserve	Use an ML model to diagnose hemodynamically relevant coronary artery disease via angiography-derived fractional flow reserve.	Pilot	No	Human comparison	AI	Maybe
						Gold standard		
Knackstedt et al. 2015.^43^	D	Ejection fraction	Create software that uses ML for efficient measurements of left ventricular volumes, ejection fraction, and biplane longitudinal strain.	Human comparison	Yes	Human comparison	AI	Maybe
Betancur et al. 2018.^44^	S	Major adverse cardiac events	Assess the added predictive value of combining clinical data with Single-photon emission computed tomography imaging via ML for major cardiac event prediction.	Human comparison	No	Human comparison	AI	Yes
Bratt et al. 2019.^45^	D	Image interpretation: CMR	Evaluate a new ML model for automated analysis of phase contrast CMR aortic flow.	Human comparison	Yes	Human comparison	AI	Yes
Bhuva et al. 2019.^46^	D	Image Interpretation: CMR	Compare automated ML with human analysis for assessing left ventricular function and mass in CMR imaging.	Human Comparison	Yes	Human Comparison	AI	Yes
Beecy et al. 2020.^47^	D	Image interpretation: Echo	Validate an ML method for automated quantification of right ventricular function using 2D Echo.	Human comparison	No	Human comparison	AI	Maybe
						Gold standard		
Ouyang et al. 2020.^48^	D	Ejection fraction	Develop and validate EchoNet-Dynamic, a DL algorithm for left ventricle segmentation, ejection fraction estimation, and cardiomyopathy assessment in echo videos.	Human comparison	Yes	Human comparison	AI	Yes
Choi et al. 2021.^49^	D	Coronary artery disease: atherosclerosis	Evaluate an AI approach for coronary artery disease risk factors via coronary computed tomography angiography, focusing on vessel morphology and stenosis.	Human comparison	Yes	Human comparison	AI	Yes
Fu et al. 2021.^50^	D	Arrhythmias: atrial fibrillation	Evaluate a wearable AI ECG recorder for atrial fibrillation detection	Human comparison	No	Human comparison	AI	Yes
Narang et al. 2021.^51^	D	Image acquirement: echo	Assess a DL software's ability to guide novices in obtaining diagnostic-quality 10-view transthoracic echos.	Human comparison	Yes	Human comparison	Joint AI-human	No
Taylor et al. 2021.^52^	D	Image interpretation: MPI	Assess an ML model for automated stress scans in myocardial perfusion patients.	Human comparison	No	Human comparison	AI	Yes
Russell et al. 2021.^5^	D	Heart failure	Assess AI quantification of B-lines in lung ultrasound images for acute heart failure patients vs human experts	Human comparison	Yes	Human comparison	Joint AI-human	Maybe
Giudicessi et al. 2021.^53^	M	Arrhythmias: congenital long QT syndrome	Assess smartphone AI's accuracy in QTc interval measurement from photoplethysmography signals vs ECG for monitoring.	Human comparison	No	Human comparison	AI	Yes
						Gold standard		
Zucker et al. 2021.^54^	D	Image acquirement: CMR	Assess image quality and performance of accelerated, free-breathing 2D cine CMR with DL reconstruction.	Human comparison	No	Human comparison	AI	Maybe
Augusto et al. 2021.^55^	D	Image interpretation: CMR	Assess automated ML-based vs expert measurement of left ventricular thickness in hypertrophic cardiomyopathy patients	Human comparison	Yes	Human comparison	AI	Yes
Yang et al. 2022.^56^	S	Image interpretation: echo	Automate echocardiographic video analysis for valvular heart diseases	Human comparison	Yes	Human comparison	AI	Maybe
Liu et al. 2022.^57^	D	Acute conditions: aortic dissection	Assess DL-based aortic dissection detection using ECG and x-rays	Human comparison	No	Human comparison	AI	Yes
Alandejani et al. 2022.^58^	D	Image interpretation: CMR	Automate right atrium area measurement in CMR imaging	Human comparison	No	Human comparison	AI	Maybe
Han et al. 2022.^59^	D	Coronary artery disease: stenosis	Assess AI's impact on inexperienced radiologists diagnosing coronary stenosis	Human comparison	No	Human comparison	AI	Yes
Alabed et al. 2022.^60^	D	Image interpretation: CMR	Assess a DL tool for CMR in pulmonary hypertension prognosis	Human comparison	Yes	Human comparison	AI	Yes
Varudo et al. 2022.^61^	D	Ejection fraction	Enable automated real-time echocardiographic left ventricular ejection fraction assessment for critically ill patients	Human comparison	No	Human comparison	Joint AI-human	No
Kim et al. 2023.^62^	S	Coronary artery disease	Improve obstructive coronary artery disease identification and reduce invasive coronary angiography costs	Human comparison	Yes	Human comparison	AI	Yes
Sartoretti et al. 2023.^63^	D	Coronary artery disease: calcium	Compare automated coronary artery calcium scoring via DL to manual measurements	Human comparison	No	Human comparison	AI	Maybe
Ajmera et al. 2023.^64^	D	Image interpretation: x-ray	Assess an AI system for detecting chest pathologies on radiographs compared to human readers. Also evaluating radiologist performance with vs without AI assistance.	Human comparison	Yes	Human comparison	AI	Maybe
							Joint AI-human	
Yang et al. 2023.^65^	D	Image interpretation: echo	Investigate the efficiency of an ML model for diagnosing patent foramen ovale via contrast transthoracic echocardiography images.	Human comparison	Yes	Human comparison	AI	Maybe
Sato et al. 2023.^66^	D	Ejection fraction: left heart abnormalities	Detect low ejection fraction, wall motion abnormalities, left ventricular hypertrophy, left ventricle and atrial dilatation.	Human comparison	Yes	Human comparison	AI	Yes
Diao et al. 2023.^67^	D	Image interpretation: CMR	Develop an automatic framework for diagnosing causes of left ventricular hypertrophy using cardiac cine images.	Human comparison	Yes	Human comparison	AI	Maybe
Liu et al. 2023.^68^	D	Heart failure	Assist physicians in assessing cardiac function to standardize echocardiographic findings and ultrasound data compatibility.	Human comparison	Yes	Human comparison	AI	Yes
Bouzid et al. 2023.^69^	D	Coronary artery disease	Compare diagnostic performance of out-of-hospital vs emergency department ECGs and evaluate AI-augmented ECG analysis for diagnosing non-ST elevation acute coronary syndrome.	Human comparison	No	Human comparison	AI	Yes
Buckler et al. 2023.^70^	S	Coronary artery disease: atherosclerosis	Evaluate ML software accuracy in determining plaque risk phenotype versus expert pathologists.	Human comparison	Yes	Human comparison	AI	Maybe
Hagio et al. 2023.^71^	D	Coronary artery disease	Evaluate DL-based attenuation correction versus non-attenuation-corrected for coronary artery disease detection, as defined by invasive coronary angiography.	Human comparison	Yes	Human comparison	AI	Yes
Mannhart et al. 2023.^72^	M	Arrhythmias: atrial fibrillation	Assess effectiveness of 5 smartwatches in detecting atrial fibrillation.	Human comparison	No	Human comparison	AI	No
Shen et al. 2023.^73^	D	Arrhythmias: atrial fibrillation	Assess a CNN's performance in diagnosing shockable arrhythmias using a novel, miniaturized automated external defibrillator.	Human comparison	Yes	Human comparison	AI	Yes
Zhou et al. 2023.^74^	T	Coronary artery disease	Use a DL model to predict guidewire crossing and patient outcomes in percutaneous coronary interventions for chronic total occlusion.	Human comparison	Yes	Human comparison	AI	Yes
						Gold standard		

AI = artificial intelligence; CMR = cardiac magnetic resonance; CNN = convolutional neural network; CT = computed tomography; D = diagnostic; DL = deep learning; ECG = electrocardiogram; Echo = echocardiography; EHR = electronic health record; M = monitoring; ML = machine learning; MPI = myocardial perfusion imaging; NR = not reported; RCT = randomized controlled trial; S = screening; T = treatment.

Details include intervention types, disease/clinical use cases, objectives, study types, whether multicenter validation occurred, study controls, study interventions, and assessment of intervention superiority or noninferiority.

The most frequent study type was comparative analysis against human experts (n = 33, 51.5%), followed by pilot implementations (n = 20, 31.3%) and RCTs (n = 11, 17.2%). Human experts were more frequently used as a study control (n = 53, 73.6%), compared to gold standard practices (n = 19, 26.4%). AI models alone were also the most common study intervention (n = 64, 79.0%), compared to joint AI-human interventions (n = 17, 21.0%). An equal number of articles were conducted in single-center and multicenter settings (n = 32, 50.0%).

### Model features and performance

Table 2 summarizes the characteristics of the included predictive models and associated data. Among the 64 articles, we identified 45 classification tasks (69.3%), 19 regression tasks (29.2%), and 1 paper did not specify the task (1.5%). Among the predictive models developed, convolutional neural networks were the most employed AI model (n = 44, 67.7%).

**Table 2:** Summary of Algorithms, Data, and Performance Metrics of the Included Studies

First Author Year	Prediction Task	Algorithm Type	Training/Test Size	Data Type	Performance Metrics	Model and/or Data Publicly Available
Blomberg et al 2021.^20^	C	NR	108,607	Audio (phone call)	SN = 0.850	No
					SP = 0.974	
Huang et al 2021.^4^	C	NR	NR	Waveform (ECG)	SN = 0.944	No
					SP = 0.985	
Yao et al 2021.^7^	C	CNN	44,959	Waveform (ECG)	AUC = 0.920	No
Luštrek et al 2021.^6^	R∗	RF	37∗	EHR (Tabular)	SN = 0.830-0.940	Yes
					MAE = 7-9	
Zisis et al 2021.^16^	C	LR	1,046∗	Image (Echo)	AUC = 0.708	No
					SN = 0.640	
					SP = 0.650	
Rushlow et al 2022.^8^	C	CNN	97,829	Waveform (ECG)	AUC = 0.930	No
					SN = 0.863	
					SP = 0.857	
Horne et al 2022.^17^	NR	RL	NR	EHR (Tabular)	NR	No
Sandhu et al 2023.^18^	R∗	CNN	866	Image (CT)	SN = 0.875-1	Yes
					SP = 0.970-1	
Yang et al 2023.^19^	R∗	CNN	NR	Image (CT)	AUC = 0.928	Yes
					SN = 0.971	
					SP = 0.750	
					MAE = −0.003-0.015	
					ICC = 0.701	
He et al 2023.^21^	R	CNN	144,184	Video (Echo)	MAD = 0.028	Yes
Šribar et al 2023.^22^	C	LR	1,334	Waveform (Arterial Pressure)	SN = 85-94%	No
					SP = 85-94%	
					AUC = 0.94-0.96	
Brennan et al 2019.^23^	C	RF	51,457	EHR (Tabular)	AUC (AI) = 0.640	No
					AUC (AI-Human) = 0.590	
Jacobsen et al 2020.^24^	C	CNN	82∗	Waveform (ECG)	AUC = 0.980	No
					SN = 0.960	
					SP = 0.990	
Liu et al 2021.^25^	C	CNN	25,002	Waveform (ECG)	AUC = 0.991-0.999	Available on request
					SN = 0.657-0.842	
					SP = 0.998-1	
Maille et al 2021.^26^	R	CNN	6,315	Waveform (ECG)	BA = 0.225-0.0113	Yes
					ICC = 0.38-0.57	
Winslow et al 2022.^27^	C	LR	162,088	EHR (Time Series)	AUC = 0.750-0.930	Yes
Bachtiger et al 2022.^28^	C	CNN	35,970	Waveform (ECG)	AUC = 0.850	Yes
					SN = 0.848	
				Image (Echo)	SP = 0.695	
Yacoub et al 2022.^29^	C	CNN	NR	Image (CT)	NR	No
Aharon et al 2022.^30^	C	NR	NR	NR	NR	No
Zhu et al 2022.^31^	C	CNN	510,566	Waveform (PPG)	SN = 0.878	No
					SP = 0.974	
Edalati et al 2022.^32^	R	CNN	Application 1 = 255∗	Image (CMR)	BA = 0.288	No
			Application 2 = 400∗		ICC = 0.780	
Noseworthy et al 2022.^33^	R∗	CNN	649,931	Waveform (ECG)	Application 1:	Available on request
					SN = 89%	
					SP = 38%	
					Application 2:	
					SN = 87%	
					SP = 38%	
					Application 3:	
					SN = 91%	
					SP = 37%	
Mor-Avi et al 2023.^34^	R	CNN	210∗	Image (Echo)	BA = −1	No
Cho et al 2023.^35^	C	NR	55,083∗	EHR (Tabular)	AUC = 0.869	Yes
Yamaguchi et al 2023.^36^	R∗	CNN	48∗	Image (Echo)	BA = −5.8	No
					AUC = 0.96	
Nurmaini et al 2023.^37^	C	CNN	1,185	Image (Echo)	ACC = 0.92	Yes
					SN = 0.92	
					SP = 0.94	
Dadon et al 2023.^38^	R	NR	NR	Image (Echo)	SN = 72.7%	No
					SP = 100%	
Celik et al 2023.^39^	C	CNN	23,000	Image (X-Ray)	SN = 90%	Available on request
					SP = 80%	
Al-Zaiti et al 2023.^40^	C	RF	4,026	Waveform (ECG)	AUC = 0.87	Yes
					SN = 86%	
					SP = 98%	
Mahajan et al 2023.^41^	C	GBT	1,016,966	EHR (Tabular)	AUC = 0.956	Available on request
					SN = 85.3%	
					SP = 91.4%	
Omori et al 2023.^42^	R∗	NR	27,000	Image (X-Ray Angiogram)	AUC = 0.90	No
					SN = 76.8%	
					SP = 94.3%	
Knackstedt et al 2015.^43^	R	ML	255∗	Image (Echo)	BA = 0.527	No
					ICC = 0.797	
Betancur et al 2018.^44^	C	LB	2,360	Image (MPI)	AUC = 0.810	No
				EHR (Tabular)		
Bratt et al 2019.^45^	C	CNN	4,345	Image (CMR)	MAE = 1.850	Yes
					BA = 0.095	
					ICC = 0.994	
Bhuva et al 2019.^46^	R	CNN	599	Image (CMR)	BA = 0.170-0.223	Yes
					ICC = 0.900-0.980	
Beecy et al 2020.^47^	R∗	CNN	7,791	Image (Echo) Image (CMR)	AUC = 0.690-0.730	Yes
					SN = 0.830-0.850	
					SP = 0.390-0.450	
					BA = 0-0.085	
					ICC = 1	
Ouyang et al 2020.^48^	C, R	CNN	10,030	Video (Echo)	AUC = 0.960	Yes
					MAE = 0.041	
					MSE = 0.053	
Choi et al 2021.^49^	C	CNN	Application 1 = 1,007,945	Image (CT)	SN = 0.800-0.909	No
			Application 2 = 1,414,877		SP = 0.970-0.998	
Fu et al 2021.^50^	C	CNN	114∗	Waveform (ECG)	SN = 0.886-0.943	No
					SP = 1	
Narang et al 2021.^51^	C	CNN	>5 million	Image (Echo)	NR	No
Taylor et al 2021.^52^	C∗	CNN	9,604	Image (MPI)	MAE = 0.050	No
Russell et al 2021.^5^	C∗	CNN	60	Image (Lung Ultrasound)	BA = 0.560-0.580	Yes
Giudicessi et al 2021.^53^	C	CNN	1,576,539	Waveform (ECG)	AUC = 0.911-0.968	No
					SN = 0.793-0.801	
					SP = 0.858-0.944	
Zucker et al 2021.^54^	R	CNN	10∗	Image (CMR)	BA = 0.034	Yes
					ICC = 0.760-0.970	
Augusto et al 2021.^55^	R	CNN	∼576,900	Image (CMR)	BA = 0.027	Yes
Yang et al 2022.^56^	C	CNN	1,335	Video (Echo)	AUC = 0.880-0.990	Yes
					SN = 0.820-0.940	
					SP = 0.880-0.940	
Liu et al 2022.^57^	C	CNN	49,071	Waveform (ECG)	AUC = 0.918	No
				Image (X-Ray)		
				EHR (Tabular)		
Alandejani et al 2022.^58^	C	CNN	10,045	Image (CMR)	AUC = 0.820-0.870	Available on request
Han et al 2022.^59^	C	CNN	10,100	Image (CT)	SN = 0.781-0.935	No
					SP = 0.579-0.825	
Alabed et al 2022.^60^	C∗	CNN	539∗	Image (CMR)	BA = 0.14	No
					ICC = 0.930-0.990	
Varudo et al 2022.^61^	R∗	CNN	NR	Image (Echo)	SN = 0.700	No
					SP = 0.980	
					MAD = 1	
Kim et al 2023.^62^	C	CNN	NR	Image (CT)	AUC = 0.610	Yes
Sartoretti et al 2023.^63^	C	CNN	100	Image (CT)	SN = 0.933	No
Ajmera et al 2023.^64^	C	CNN	1.5 million	Image (X-Ray)	AUC (AI) = 0.912	No
					SN (AI) = 0.884	
					SP (AI) = 0.885	
					AUC (AI-Human) = 0.879	
					SN (AI-Human) = 0.851	
					SP (AI-Human) = 0.895-0.919	
Yang et al 2023.^65^	C	CNN	200∗	Image (CT)	SN = 0.780	Available on request
				Image (Echo)	SP = 0.750	
Sato et al 2023.^66^	C	CNN	229,439	Image (Echo) Waveform (ECG)	Application 1:	No
					CNN: ACC = 78.3%, SN = 68.9%; SP = 93.3%	
					Lead 1 cardiologist: ACC = 65.6%, SN = 53.7%; SP = 78.6%	
					12-lead cardiologist: ACC = 69.1%, SN = 60.3%; SP = 80.0%	
					Application 2:	
					CNN: ACC = 68.3%, SN = 40.0%; SP = 96.7%	
					Lead 1 cardiologist: ACC = 55.6%, SN = 32.2%; SP = 79.1%	
					12-lead cardiologist: ACC = 56.9%, SN = 39.1%; SP = 73.6%	
Diao et al 2023.^67^	C	RNN	302∗	Image (MRI)	AUC = 0.848-0.983	Available on request
		SVM				
Liu et al 2023.^68^	R∗	CNN	8,976 images	Image (Echo) Video (Echo)	AUC = 1	Yes
			10,085 videos			
Bouzid et al 2023.^69^	C	RF	1,699	Waveform (ECG)	Application 1:	No
					SN = 62%-86%	
					SP = 71%-80%	
					PPV = 29%-39%	
					NPV = 92%-97%	
					Application 2:	
					SN = 23%-50%	
					SP = 82%-89%	
					PPV = 21%-39%	
					NPV = 87%-90%	
Buckler et al 2023.^70^	C	CNN	408 (23∗)	Image (CT)	AUC = 0.95-0.99	No
Hagio et al 2023.^71^	C	CNN	11,532	Image (MPI)	AUC = 0.752	Yes
					SN = 79.7%	
					SP = 56.2%	
Mannhart et al 2023.^72^	C	NR	NR	Waveform (ECG)	SN (smartwatch) = 58%-85%	No
					SP (smartwatch) = 69%-79%	
					SN (AI) = 87%-95%	
					SP (AI) = 93%-98%	
Shen et al 2023.^73^	C	CNN	26,464	Waveform (ECG)	AUC = 0.998	Available on request
					SN = 97.9%	
					SP = 99.0%	
Zhou et al 2023.^74^	C	TR	534∗	Image (CT)	Application 1:	Yes
					AUC = 0.96	
					SN = 94.4%	
					SP = 94.3%	
					Application 2:	
					AUC = 0.96	
					SN = 96.6%	
					SP = 95.3%	

2D = 2-dimensional; ACC, accuracy; AUC = area under receiver-operating curve; BA = Bland-Altman score; C = classification; GBT = gradient boosted tree; ICC = intraclass correlation; R = regression; LB = LogitBoost; LR = logistic regression; MAD = mean absolute difference; MAE = mean absolute error; MSE = mean squared error; MRI = magnetic resonance image; NPV, negative predictive value; PPG = photoplethysmogram; PPV = positive predictive value; RF = random forest; RL = reinforcement learning; RNN = recurrent neural network; SVM = support vector machine; SN = sensitivity; SP = specificity; TR = transformer; other abbreviations as in Table 1.

C∗: Classification model used for regression task; R∗: Regression model used for classification task.

For 'Training/Test Size', values with an asterisk (∗) refer to the number of patients, while the remainder refers to the number of data instances.

Details include prediction task, algorithm type, training/test size, data type, performance metrics and whether the model and/or data is publicly available. Footnotes are used to clarify data collection nuances. Model training data size is listed primarily by sample count, with studies only reporting number of patients used for model training marked with an asterisk. Likewise, we use asterisk when AI models are repurposed beyond their training objectives to address distinct clinical needs, leading to a misalignment between their training prediction task type and application (eg, a regression model used in classification applications).

**Figure 4:**
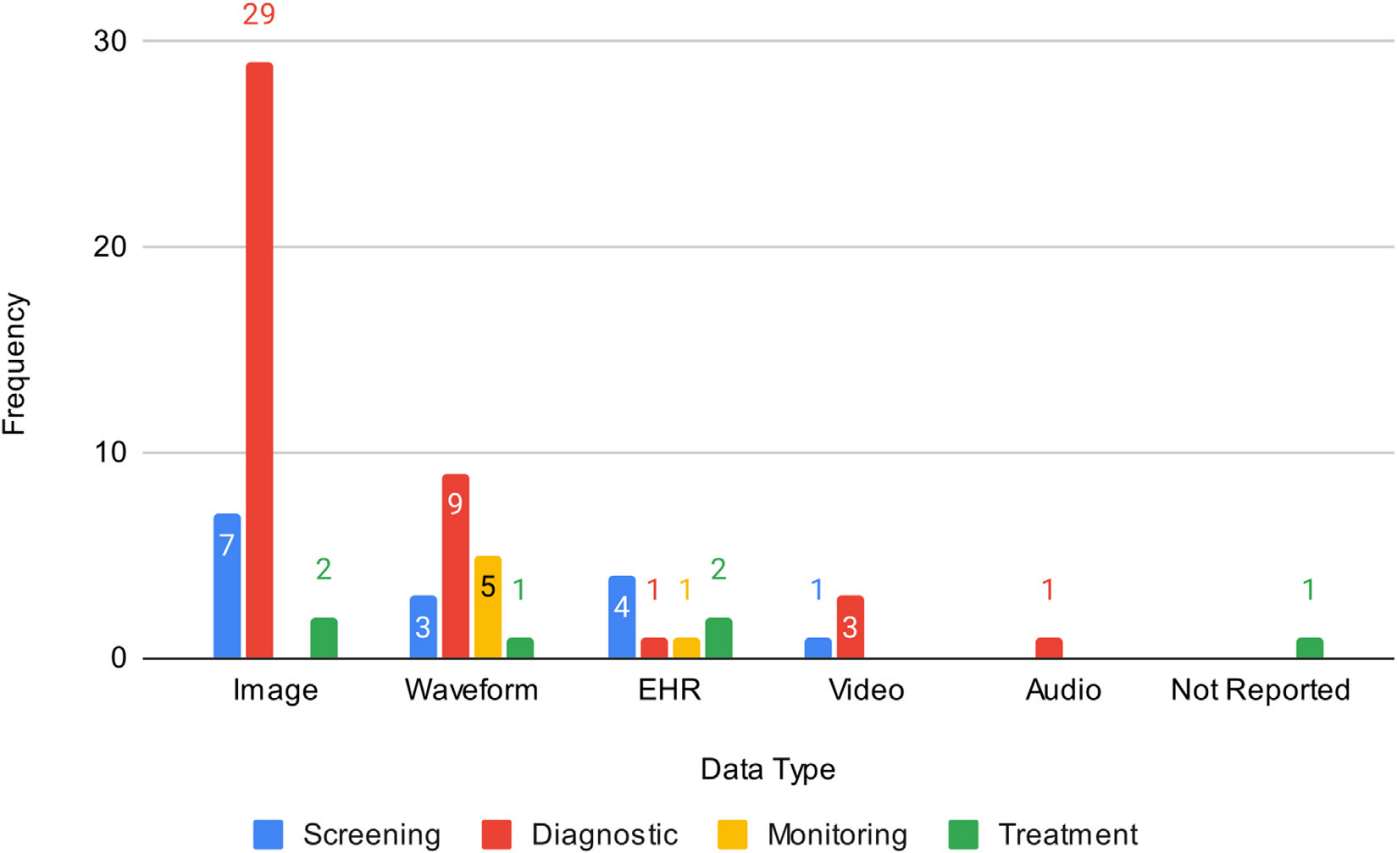
**Frequency of Data Types by Intervention Categories** The number of studies by data type and intervention type: Screening (evaluation of potentially at-risk asymptomatic patients), diagnosis (analysis of symptomatic patients), monitoring (continuous assessment of patients to predict adverse events), and treatment (enhancing management and therapeutic care approaches). EHR = electronic health record.

Most studies use images as their primary data type (n = 38, 54.3%) to develop the AI intervention (Figure 4), followed by waveforms (n = 18, 25.7%). As expected, the size of the training data set used varied drastically across different publications (Table 2). Among those reporting the individual training samples, the median was 11,532 (IQR: 3,193-153,136). For those reporting the number of patients in the training data, the median was 232.5 (IQR: 73.5-433.5). The median number of individual training samples used across data types were image (10,045 [IQR: 1,345-229,439]), waveform (35,970 [IQR: 6,315-97,829]), EHR (162,088 [IQR: 106,772.5-716,648]), and video (10,058 [IQR: 7,856.25-43,609.75]). The 1 study used audio data and reported 108,607 samples.

The median model performance for the classification tasks was 0.918 (IQR: 0.845-0.956) for AUC, 0.860 (IQR: 0.805-0.907) for sensitivity, and 0.906 (IQR: 0.801-0.979) for specificity. For regression tasks, the reported comparable metrics include measures of agreement. Specifically, there was a median normalized BA score of 0.043 (IQR: 0.027-0.197) and a median ICC of 0.797 (IQR: 0.741-0.903).

Interestingly, we identified 2 articles^23^^,^^64^ reporting the use of AI models in comparison to joint human-AI decision-making. Both studies showed that joint human-AI decision-making resulted in lower AUCs of 0.59 and 0.88, respectively, compared to AUCs of 0.64 and 0.91 for the AI model alone. While not reporting specific prediction performance metrics, Blomberg et al^20^ also reported joint AI-human decision-making was inferior to the AI model alone (eg, slower diagnosis, fewer correct diagnoses). Nonetheless, joint human-AI decision-making still outperformed the respective study’s controls.

The reported models demonstrated limited reproducibility and open access, with 22 (34.4%) of the publications sharing the trained model or model code. Furthermore, 8 (12.5%) offered data availability only upon request, and 34 (53.1%) publications lacked the availability of both models and data.

### Study outcomes

Among the 64 included articles, 44 (68.7%) reported either clinical or operational benefits of the AI intervention compared to the study’s control. In contrast, 17 records (26.6%) reported conflicting metrics (eg, worse predictive performance but significantly faster task completion), and 3 records^51^^,^^61^^,^^72^ (4.7%) indicated AI interventions performed worse than the study controls. The 3 articles with poor performance involved AI models used for echocardiogram acquirement, ejection fraction assessment, and atrial fibrillation detection.

**Figure 5:**
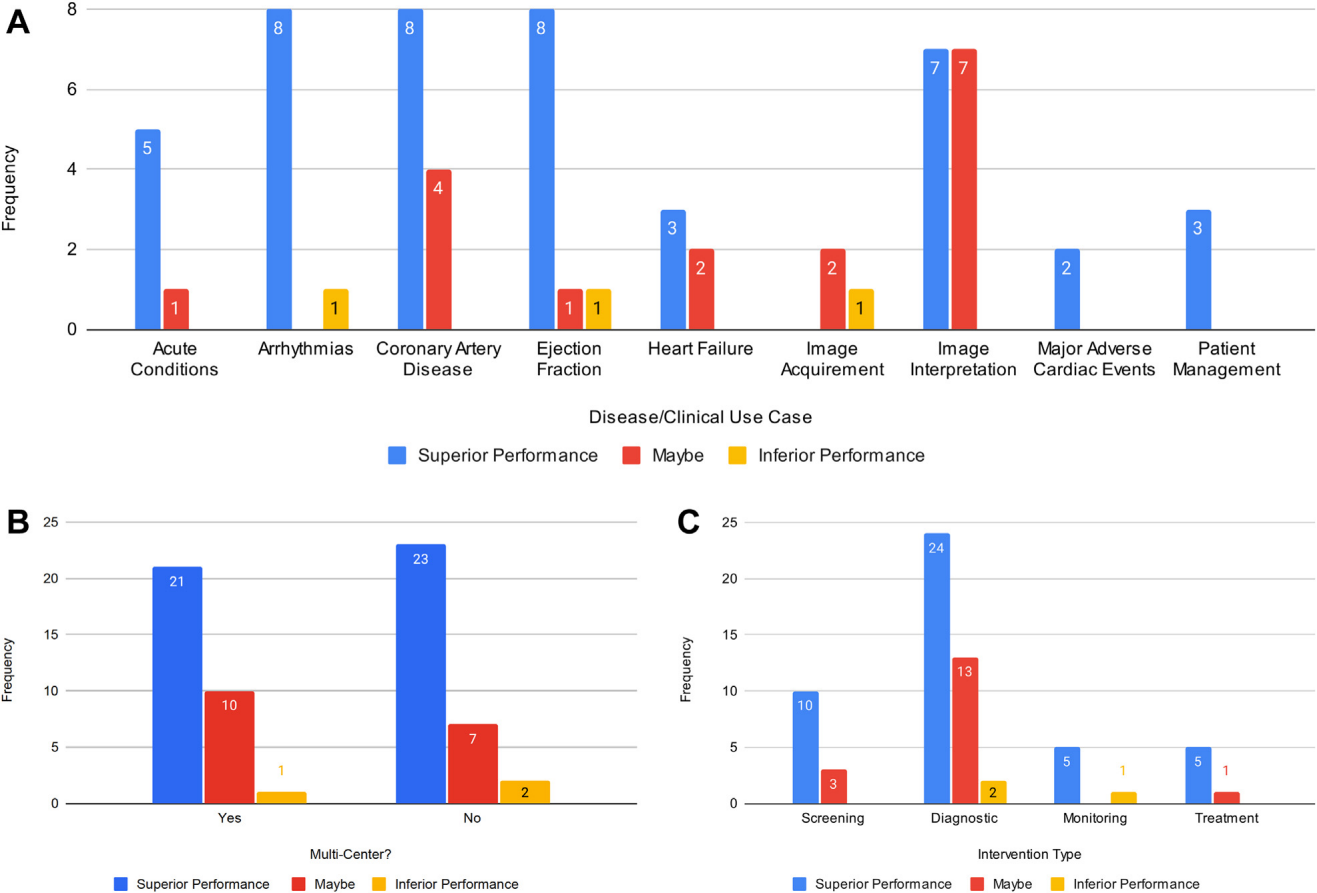
**Frequency of Intervention Superiority/Noninferiority by Different Categories** (A) Clinical use case. (B) Multicenter. (C) Intervention type. A displays the frequency by clinical use cases. B demonstrates the frequency based on whether the AI model is being validated via a multicenter data set. C illustrates the frequency based on intervention type.

Figure 5 summarizes the outcomes of the studies (ie AI-based intervention superiority or noninferiority compared to the control) across clinical use case (Figure 5A), multicenter validation (Figure 5B), and intervention type (Figure 5C). Among clinical use cases (Figure 5A), AI interventions focused on patient management, major adverse cardiac events, and arrhythmias almost always had positive study results. Studies focused on image acquirement, image interpretation, and heart failure reported the highest proportion of mixed or negative study results. Intervention successes were similar in single-center and multicenter studies, signaling potential AI model generalizability (Figure 5B). Among intervention types, screening and treatment were the only categories that did not report inferior study outcomes (Figure 5C). Additionally, human expert comparison studies yielded the highest amount of mixed or negative results, suggesting a continued need to evaluate AI models before RCTs and pilot implementations (Central Illustration).

**Central Illustration:**
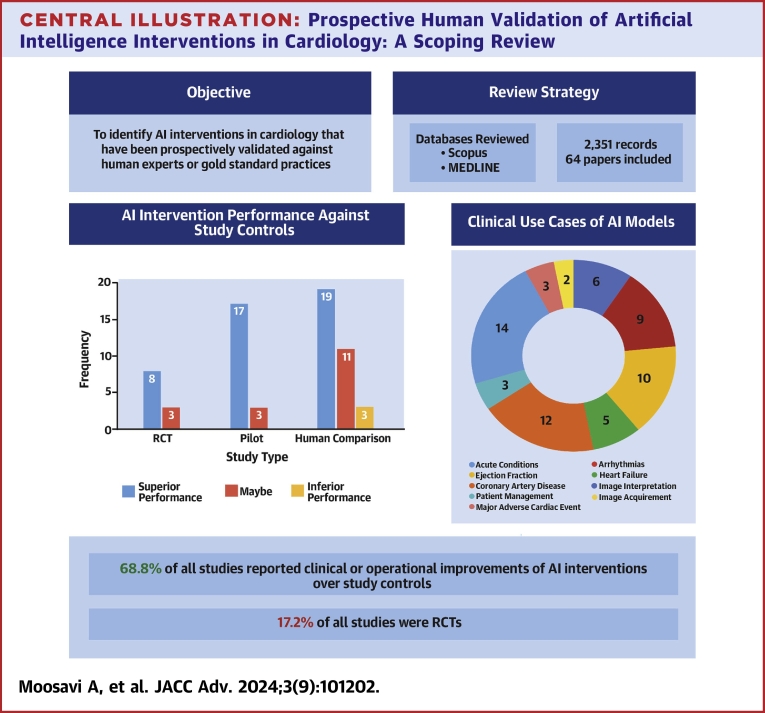
**Prospective Human Validation of Artificial Intelligence Interventions in Cardiology: A Scoping Review** AI = artificial intelligence; other abbreviation as in Figure 2.

## Discussion

In our review, we observed an increasing trend in the prospective human validation of AI models over time. Most studies showed clinical or operational (eg, accelerating decision-making processes and decreasing time of task completion^20^^,^^21^^,^^29^) advantages of AI-based interventions compared to their human counterparts. This is especially true for uses focused on screening, treatment, arrhythmias, coronary artery disease, ejection fraction, and image interpretation, as well as for models using image and electrocardiogram data. Despite these promising results, a significant gap emerges with the evaluation of AI models in real-world settings, with only 11 RCTs^4^^,^^6^, ^7^, ^8^^,^^16^, ^17^, ^18^, ^19^, ^20^, ^21^, ^22^ identified in this review.

This review also identified multiple opportunities for AI innovation in cardiology. Further study of AI applications for monitoring^4^^,^^27^^,^^53^ and treatment^7^^,^^17^^,^^26^ is warranted, given the few but successful studies demonstrating the value of AI where continuous human monitoring is impractical due to fatigue and where efficiently making treatment decisions is challenging. Similarly, additional exploration of use cases with fewer works (ie, image acquirement, major adverse cardiac events, patient management) as well as mixed study outcomes (ie, heart failure, ejection fraction^5^, ^6^, ^7^, ^8^^,^^16^^,^^21^^,^^28^^,^^43^^,^^48^^,^^61^) is needed. The varied outcomes in human comparison studies suggest the need for continued AI validation, specifically against human experts before RCTs and pilot studies. Novel modeling techniques (eg, multimodal learning^75^) should also be further explored to improve impact. Notably, our search did not yield generative AI model studies, despite its validation in various health care areas.^76^ This underscores the need for prospectively validating generative AI in cardiology. Furthermore, innovative data sources (eg, EHRs, video, audio, free-text, genetic, biomarker data) should be used, given their significant potential to improve predictive accuracy and patient outcomes.^75^^,^^77^

The real-world clinical and operational impact of AI tools hinges on their adoption by health care professionals,^12^ a topic that is rarely assessed. Only Rushlow et al^8^ report the considerable variation in the adoption of AI recommendations and its subsequent impact on patient care. They find that increased adoption of an AI tool in primary care nearly doubled the detection rates of low ejection fraction. Metrics of AI adoption are not explicitly explored in other RCTs within our review. Notably, Yao et al^7^ indirectly measure the impact of AI adoption in an RCT, reporting provider access to AI tools nearly doubles the diagnostic care delivered. The explicit evaluation of adoption in future RCTs is essential for guiding AI tool development and addressing barriers to adoption (eg, algorithmic trust, technological literacy, interface design, AI use across provider expertise).^8^^,^^78^

The optimal use of AI tools by humans also remains underexplored in current studies. Human-AI collaboration has the potential to benefit decision-making in ways that cannot be accomplished by exclusively using one or the other alone.^13^^,^^79^^,^^80^ An effective partnership between cardiologists and AI should leverage the distinctive capabilities of each, such as the contextual knowledge of cardiologists that is not currently incorporated as features into the majority of existing AI tools^79^^,^^80^ and the data-processing efficiency and pattern recognition of AI models. While most studies measured AI and joint AI-human interventions against humans alone,^22^^,^^24^^,^^26^^,^^34^^,^^36^ only 3 studies compared AI against joint AI-human performance. Specifically, Ajmera et al,^64^ Brennan et al,^23^ and Blomberg et al^20^ found that the AI model alone had superior performance compared to joint AI-human approaches for x-ray pathologies prediction tasks, predicting cardiac complications following surgical procedures and identifying out-of-hospital cardiac arrests, respectively. We hypothesize that this performance gap arises from a lack of AI model adoption among providers, especially those with higher expertise.^78^ This reluctance is likely due to low algorithmic trust, stemming from insufficient evidence supporting AI model use.^81^ Importantly, over-reliance on AI models in high-risk settings or with less reliable models can be harmful.^78^ Balancing trust in AI models as well as enhancing model development and operationalization is crucial to improve the performance of human-AI teams in practice.

Regarding the design of future prospective validation studies, researchers should consider the merits of prospective validation across both single-center^82^ and multicenter^83^ settings (ie, consider the limitations of multicenter validation for AI use in practice compared to recurring local validation) as well as pragmatic trials^84^ for informing AI tool implementation, evaluating and updating models over time to mitigate data drift (ie, the deterioration of AI model performance due to changes in data patterns over time, such as with patient demographics or operational practices),^85^ measuring impact on mortality as opposed to intermediate end points to gauge real-world effectiveness,^86^ standardizing descriptions and sharing practices for AI models and data,^87^^,^^88^ and adherence to prospective AI evaluation guidelines.^87^, ^88^, ^89^ These practices will accelerate AI research and adoption for improving cardiovascular care.^13^^,^^14^

### Study Limitations

First, despite following search protocols, it is possible that relevant studies were not identified due to the different keywords that could be used to describe human expert validation. Second, our findings, current as of 2023, may not include the latest developments in AI cardiology due to the rapidly evolving AI landscape. Third, study quality or risk of bias were not assessed, which might affect outcome interpretation. Nevertheless, we reported indicators of quality (eg, data set size). Finally, publication bias, where positive results are more likely to be published than negative ones, could impact the findings.

## Conclusions

Our review highlights the potential of AI in cardiology, notably in arrhythmias, coronary artery disease, ejection fraction, and image interpretation uses, demonstrating operational and clinical advantages over human experts. Despite these advancements, there is still a critical need for real-world validation to better determine the impact of AI in practice. The use of diverse data types, beyond medical images and waveforms, may also enhance the impact of AI interventions. Furthermore, we underscore the importance of joint human-AI decision-making, suggesting that future research prioritize combining the strengths of AI and human experts.

Perspectives **COMPETENCY IN SYSTEMS-BASED PRACTICE/PATIENT CARE:** AI-based models have demonstrated advantages over human experts and current practices for specific use cases in cardiology via prospective validation studies.

**TRANSLATIONAL OUTLOOK:** Despite reported successes of AI-based interventions, effective clinical translation of AI in cardiology requires more extensive real-world prospective validations assessing their efficacy and integration into clinical practice, including the investigation of the adoption of AI tools and joint human-AI decision-making.

## Funding support and author disclosures

The authors have reported that they have no relationships relevant to the contents of this paper to disclose.
