# Supplementary material for: Prospective Human Validation of Artificial Intelligence Interventions in Cardiology: A Scoping Review
Source: JACC Adv. 2024 Aug 28;3(9):101202. doi: 10.1016/j.jacadv.2024.101202 (PMC11450923; doi:10.1016/j.jacadv.2024.101202)
Supplement: Supplementary Materials [file mmc1.docx]

#

# **Supplemental Methods**

## **Search Strategy and Selection Criteria**

Both MEDLINE and Scopus online databases were systematically searched by S.H. and a professional science and engineering librarian. The search was limited to include all English published papers between January 2015 and December 2023. As multiple papers focused on prospective human expert and real-world model validation were accompanied by prior paper detailing the retrospective training and testing of the Artificial Intelligence (AI) model, backwards citation searches were performed during the data extraction stage when necessary to obtain the training papers. We used the following search queries in MEDLINE and Scopus:

MEDLINE Search Terms

| 1. | exp *machine learning/ or exp *neural networks, computer/ |
| --- | --- |
| 2. | exp Cardiology Service, Hospital/ or exp Cardiology/ or exp Coronary Disease/ or exp Myocardial Infarction/ or  exp Cardiovascular Diseases/ |
| 3. | ("machine learning" or "deep learning" or "artificial intelligence" or "neural network*").tw. |
| 4. | ("cardiology" or "cardiovascular").tw. |
| 5. | prospective*.tw,kf. and ((validat* or verif*).tw,kf. or "validation study"/) |
| 6. | ("clinical trial" or "controlled trial" or multicent* or multi-cent*).tw,kf. |
| 7. | clinical study/ or multicenter study/ or validation study/ |
| 8. | (1 or 3) and (2 or 4) and (5 or 6 or 7) |
| 9. | limit 8 to "review articles" |
| 10. | 8 not 9 |
| 11. | limit 10 to english language |
| 12. | limit 11 to yr="2015 - 2023" |

Scopus Search Terms

( TITLE-ABS-KEY ( "machine learning" OR "deep learning" OR "artificial intelligence" OR "neural network" ) AND TITLE-ABS-KEY ( "cardiology" OR "cardiovascular" OR "cardiovascular disease" OR "myocardial" OR "heart failure" OR "heart attack" OR "heart disease" OR "coronary" OR "cardiac" ) AND TITLE-ABS-KEY ( ( "prospective*" AND ( validat* OR verif* ) ) OR "clinical trial" OR "controlled trial" OR ( multicent* OR multi-cent* ) ) ) AND PUBYEAR > 2014 AND ( LIMIT-TO ( DOCTYPE , "ar" ) OR LIMIT-TO ( DOCTYPE , "cp" ) OR EXCLUDE ( DOCTYPE , "re" ) ) AND ( LIMIT-TO ( LANGUAGE , "English" ) )

Search Strategy and Screening Details

Relevant studies were imported into the Covidence Systematic Review management program (<https://www.covidence.org/>) for abstract and full text screening based on our inclusion and exclusion criteria. For the title and abstract screening, two votes from independent reviewers (S.H., N.T., B.M.) were required for inclusion or exclusion. Conflicts were resolved by a third independent reviewer (C.S.). Full text screening followed the same process, where two votes from two different reviewers (S.H., N.T., B.M., A.M., M.V.) were required. Conflicts were resolved by a third independent reviewer (C.S.).

## **Data extraction**

Five reviewers (S.H., C.S., B.M., A.M., M.V.) performed extraction on the selected papers and related papers identified via backwards citation searching describing the AI intervention being validated. When a reviewer encountered challenges in extracting data, such as hesitancy or difficulty obtaining information, a second independent reviewer stepped in to assist and ensure the thorough completion of the data extraction process.

Regression Task Performance Metrics

For regression tasks, data on Bland-Altman plots and the Intraclass Correlation Coefficients were collected, which measure agreement and reliability. Bland-Altman metrics were primarily reported in the form of plots. To achieve a consistent understanding of this metric across articles, we estimated and normalized it from the plots or, when available, from the tables. We measured the absolute value of the ratio of the bias to the range of agreement to enable comparison between studies that use different units in the Bland-Altman metrics. If the exact numbers were not mentioned, we estimated the standardized value from the plots.

Data Collection Footnotes

Due to the heterogeneity of the reported data, we also denoted several footnotes to specify how the data was collected. First, we prioritized listing model training data size with respect to the samples used to train the model (e.g., number of ECGs used to train the model). However, in cases where the number of samples were not available, we instead report the number of patients that the data was derived from and denoted these entries with an asterisk. Second, AI models could be repurposed beyond their training objectives to address distinct clinical needs, leading to a misalignment between their training prediction task type and application. For example, image segmentation problems are categorized as classification tasks, but the end clinical application often relates to a measurement of a continuous variable. Similarly, regression models could be applied in scenarios where outputs are interpreted through thresholding to make categorical decisions. In cases where the model was repurposed, we marked the prediction task type value with an asterisk.

## **Statistical Analysis**

We note that the total counts for some variables (e.g., study control or data type) may exceed the total number of extracted papers (64). This discrepancy arises because some papers may be classified into multiple categories (e.g., employing both human and gold standard treatment programs as their study control; utilizing various types of data in their model). Additionally, when summarizing the performance metrics of regression models, we focused on the normalized Bland-Altman score and the Intraclass Correlation Coefficient. This is because other metrics like Mean Absolute Deviation, Mean Absolute Error, and Mean Squared Error can be reported in a variety of units, preventing direct comparison. Nonetheless, all individual regression performance metrics for each paper are reported.
